# Removal of Lens for Extreme Myopia

**Published:** 1899-01-21

**Authors:** 


### REMOVAL OF LENS FOR EXTREME MYOPIA.

MR. BRUDENELL CARTER, writing in the *Lancet*, draws attention to the treatment of extreme myopia by the removal of the transparent crystalline lens, an operation which has been done in a considerable number of cases abroad, but has not been received with any great amount of favour in this country. As he points out, however, the conditions of the problem are much altered by the much greater safety with which the lens can be removed at the present day than was formerly the case. It is only since the risks which used to be inseparable from such operations have been practically extinguished by the introduction of aseptic methods of proecdure, that the suggestion to remove the lens for the relief of such a condition as myopia could be seriously entertained. In view, however, of the pitiable condition to which some patients with high degrees of myopia are reduced, an operation of this kind, done under modern conditions, is perfectly justifiable, as is well illustrated by the cases related by Mr. Carter. The second of these is the most striking. In it the patient had a progressively increasing myopia, with astigmatism, of such degree that finally she had to use for the right eye −18·0 spherical and −6·0 cylindrical, and for the left eye −12·5 spherical and −6·0 cylindrical; but even with these glasses it was impossible to obtain half the normal acuity of vision, and the condition of the patient was very miserable. She once dropped her glasses in her own bedroom, and could only find her way to the door by feeling for it round the walls. The lenses were therefore needled and afterwards evacuated, with the result that her distant vision became better than it had ever been before, even with glasses, and was raised to normal by the use of +1·5 spherical, adding for the right eye +0·5 cylindrical, and for the left +1·5 cylindrical, axes vertical; while with +4·0 spherical and the same cylindrical she could read "brilliant" type with perfect facility, which she has since continued to do without discomfort or fatigue. There can be no reasonable doubt that an eye with above 12 dioptres of myopia is likely to work better without the lens.

